# SARS-CoV-2 Receptor Binding Domain as a Stable-Potential Target for SARS-CoV-2 Detection by Surface—Enhanced Raman Spectroscopy

**DOI:** 10.3390/s21134617

**Published:** 2021-07-05

**Authors:** Chawki Awada, Mohammed Mahfoudh BA Abdullah, Hassan Traboulsi, Chahinez Dab, Adil Alshoaibi

**Affiliations:** 1 Department of Physics, College of Science, King Faisal University, P.O. Box 400, Al-Ahsa 31982, Saudi Arabia; adshoaibi@kfu.edu.sa; 2 Department of Biological Sciences, College of Science, King Faisal University, P.O. Box 400, Al-Ahsa 31982, Saudi Arabia; mbaabdullah@kfu.edu.sa; 3 Department of Chemistry, College of Science, King Faisal University, P.O. Box 400, Al-Ahsa 31982, Saudi Arabia; htraboulsi@kfu.edu.sa; 4 Département de Chimie, Université de Montréal, Campus de MIL, Montréal, QC H2V 0B3, Canada; chahinez.dab@umontreal.ca

**Keywords:** SERS, SARS-COV-2 receptor binding domain, silver/gold nanostructures, BSA

## Abstract

In this work, we report a new approach for detecting SARS-CoV-2 RBD protein (RBD) using the surface-enhanced Raman spectroscopy (SERS) technique. The optical enhancement was obtained thanks to the preparation of nanostructured Ag/Au substrates. Fabricated Au/Ag nanostructures were used in the SERS experiment for RBD protein detection. SERS substrates show higher capabilities and sensitivity to detect RBD protein in a short time (3 s) and with very low power. We were able to push the detection limit of proteins to a single protein detection level of 1 pM. The latter is equivalent to 1 fM as a detection limit of viruses. Additionally, we have shown that the SERS technique was useful to figure out the presence of RBD protein on antibody functionalized substrates. In this case, the SERS detection was based on protein-antibody recognition, which led to shifts in the Raman peaks and allowed signal discrimination between RBD and other targets such as Bovine serum albumin (BSA) protein. A perfect agreement between a 3D simulated model based on finite element method and experiment was reported confirming the SERS frequency shift potential for trace proteins detection. Our results could open the way to develop a new prototype based on SERS sensitivity and selectivity for rapid detection at a very low concentration of virus and even at a single protein level.

## 1. Introduction

In late December 2019, a patient in Wuhan, China, was diagnosed with pneumonia of a novel coronavirus later named Severe Acute Respiratory Syndrome Coronavirus 2 (SARS-CoV-2) [1]. The disease caused by SARS-CoV-2 was called Coronavirus Diseases 2019. The virus has spread worldwide rapidly, and the disease was declared a pandemic by WHO on 11 March 2020. SARS-CoV-2 is a positive-sense single-stranded RNA virus. The genomic RNA is approximately 30 Kb in length and acts as a messenger RNA (mRNA) to immediately translate viral proteins. SARS-CoV-2 genome includes 14 open reading frames (ORFs). There are two ORFs, ORF1a and ORF1b, which occupy the 5′ two-thirds of the genome and encode the nonstructural proteins (NSPs). These proteins act as complex replicase machinery [2]. On the other end, the 3′ one-third of the genome encodes four structural proteins; spike (S), envelope (E), membrane (M), and nucleocapsid (N) proteins. The structural proteins represent the components of mature virus structure. Spike protein (S) has an essential role in virus binding to host cell surface receptors [3]. It is highly conserved among all human coronaviruses (HCoVs) [4]. The S protein consists of two subunits, S1 and S2. The S protein binds to the host receptor angiotensin-converting enzyme 2 (ACE2) via the receptor-binding domain (RBD, ~200 amino acid) in the S1 subunit and fuses to the host cell via the S2 subunit [5]. Thus, RBD is the intrinsic key for the binding of SARS-CoV-2 by ACE2. RBDs have unique and structurally conserved conformational-epitopes, making them potential therapeutic targets [6]. It has been shown RBD as a target for SARS-CoV-2 detection and RBD-based antibody assays for serology [7,8,9].

Since the emergence of SARS-2, the world has been fighting to control disease spread by applying several early diagnostic techniques [10]. Real-time reverse-transcription polymerase chain reaction (rRT-PCR) is the most widely used technique for SARS-CoV-2. However, the rRT-PCR procedure requires time (it takes around 3 h, including sample preparation), reagents and well-trained personnel. Moreover, RNA sample preparation can affect the diagnostic output. Therefore, there is a great demand for a new method that allows early detection of the virus.

Raman spectroscopy (RS) is vibrational spectroscopy able to figure out chemical bonds via photon scattering, but the generated signals are extremely weak. To overcome such limitation of RS, surface-enhanced Raman spectroscopy (SERS) is used as an option to detect trace materials by taking the benefit from the enhanced electromagnetic field resulted from exciting localized surface plasmon resonances at nanostructured metallic surfaces. 

SERS technique has been intensively used in several biological analyses such as biological imaging [11], bacteria, and virus detections [12,13]. SERS has been proven efficient as well as a self-assembled quantum scale template for the development of an ultra-high noble metal-free sensor for biomolecular detection where the intracellular structures of different cell lines were unveiled [14]. Additionally, organic-based materials showed important SERS biosensing for live monitoring of the biomolecular changes within fibroblast and cancer cells [15]. Recently, the SERS technique was used to detect COVID-19 in the saliva of patients. The SERS measurements were conducted on a glass slide covered with commercially available aluminum foil. This Raman-based technique allowed the signal discrimination on samples collected from COVID-19 patients with accuracy, sensitivity, precision, and specificity of more than 95% [16]. In another recent work, Zhang et al. showed the possibility of using the Raman technique to detect the presence of SARS-CoV-2 in water. The method was based on using a silver-nanorod SERS array functionalized with cellular receptor angiotensin-converting enzyme (ACE2) [17]. Despite these promising results toward the development of SERS-based biosensors, further investigations should be performed. For instance, although it is highly considered for vaccines and therapeutic drug development, there are no reported studies on the specific detection of SARS-COV-2-RBD using the SERS technique. Additionally, controlling several parameters such as the type of substrates, the detection limits, and proteins’ fingerprints should help to develop biosensors able to detect the virus with accuracy, specificity, and high sensitivity in a short time. Finally, the novelty here consists of the study for the first time of a small protein such as RBD with a size comparable to the localized surface plasmon polaritons (LSPPs). 

**Figure 1:**
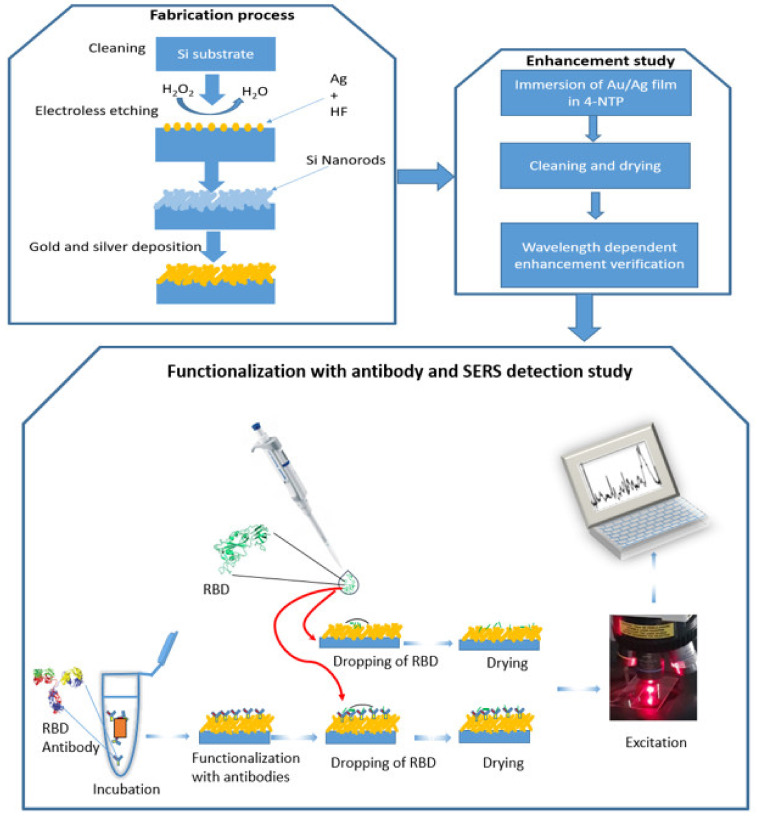
A schematic representation of three processes: 1—fabrication process, 2—enhancement study, and 3—functionalization with antibody and RBD detection by SERS.

In this work, we performed SERS measurement on Au/Ag nanoparticles deposited on silicon nanorods. We developed SERS substrate based on these nanostructures by using electroless etching and sputtering techniques, see Figure 1. The substrate showed a significant optical enhancement by using 4-NTP as a probe molecule. Two detection approaches are performed (see Figure 1: The first approach is to identify RBD protein on the surface of the substrate without any binding agent and the second one is by using SARS-CoV-2-RBD antibody). For the direct one, a detection limit with an order of magnitude of 1 pM has been achieved and during 3 s. On the other hand, using RBD antibodies confirmed the specificity of the SERS technique to identify RBD from other proteins. Finally, we simulate a 3D model of silicon nanorods surrounded by silver nanospheres using finite element method-based COMSOL software to quantitatively determine the near-field enhancement factor and estimate the plasmon frequency shift in the same experimental conditions. This technique will pave the way to detect the COVID-19 virus in a short time and with no pre-request treatment. Additionally, it can be used to study the specificity of RBD antibodies.

## 2. Experimental Part

### 2.1. Substrate Fabrication

Different steps have been carried out in order to fabricate the SERS substrates, 1—cleaning, 2—electroless etching, and 3—sputtering of silver and gold nanoparticles. A p-doped silicon substrate with a resistivity of 7 Ω·cm is cleaned with acetone, ethanol, and deionized water. After that, it is rinsed in Piranha solution (70% H_2_SO_4_/30%H_2_O_2_) in order to remove the organic residues rinsed again with deionized water. The substrate was then immersed in an aqueous solution of fluoride acid (HF) and AgNO_3_ (2 M/0.02 M) for 30 min. The etched Si substrate was dipped again in an aqueous solution of HCl/HNO_3_/H_2_O overnight in order to remove the silver residues. Then, we deposited silver nanoparticles and gold nanoparticles by using a DC magnetron sputtering system (AJA Orion 3-UHV) with the following conditions: 10 mTorr, 120 W at ambient temperature. We deposited firstly a thin layer of silver NPs during 160 s and secondly a very thin layer of gold nanoparticles during 30 s. Two targets from Labtech Inc. (Heathfield, UK) were used. The distance between the sample and the target is maintained at 15 cm. The sample was rotated azimuthally in order to disperse the particles homogeneously. In addition, the direction of the particle deposition is directed along the normal to the surface of the substrate. 

### 2.2. Instrumentation

A Jeol, JSM 7000 series Scanning electron microscope (Jeol Ltd., Tokyo, Japan) was used to obtain the morphological micrographs of films at 15.0 kV scan voltage. Raman spectra were collected using a confocal Raman microscope (LabRAM HR800, Horiba Scientific, Villeneuve-D’Ascq, France) in a backscattering geometry with a spectral resolution of 1.25 cm^−1^ at ambient temperature. A laser diode with λ = 785 nm with an output power of 300 mW and a He–Ne Laser of λ = 632.8 nm with an output power of 2 mW was used for the Raman measurement. An objective of 50×, a grating of 600 L/mm, and a selected power of 50–100 µW were used for SERS measurements. For far-field measurement, it was measured by focusing the laser spot into the drop during 150 s and with a selected power of 1 mW. The lower power was chosen in order to prevent any damage to the biomolecules or denaturation.

### 2.3. Preparation of Protein

SARS-CoV-2 RBD protein was obtained from Sino Biological company (catalogue number: 40592-V08B, Beijing, China), while BSA was obtained from Sigma-Aldrich (CAS Number 9048-46-8, St. Louis, MO, USA). Both proteins were delivered in lyophilized form. For RBD protein, the stock was reconstituted by adding sterile water (400 µL) to the vial to prepare a 0.25 mg/mL stock solution. For BSA, the stock was reconstituted by adding sterile water to prepare 1 mg/mL stock solution. Samples were prepared by diluting stock solution by sterile water to a final concentration of 10^−12^ M. The final concentration was chosen based on the reported viral load in biofluid [18,19]. SARS-CoV-2 Spike Neutralizing Antibody was obtained from Sino Biological company (catalogue number 40592-MM57) in a liquid form.

## 3. Results and Discussion

### 3.1. SEM and EDX

**Figure 2:**
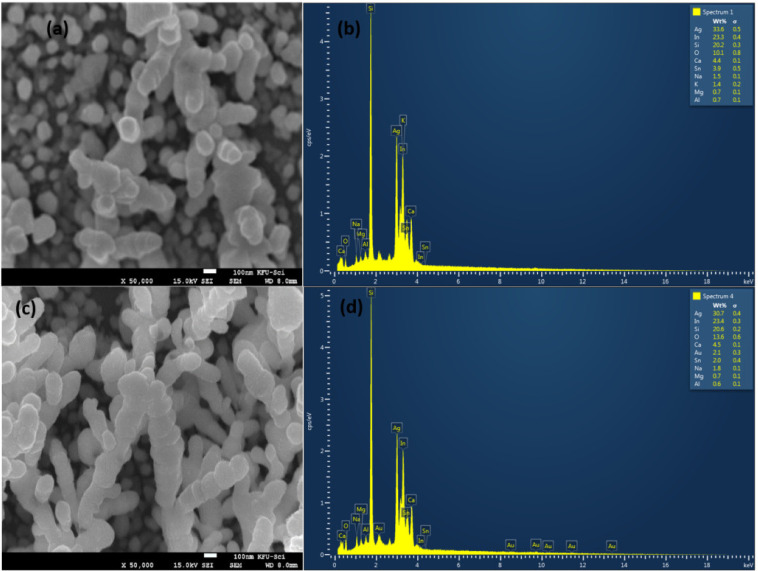
SEM images and EDX spectra of pure silver nanoparticles and silver/gold nanoparticles covering silicon nanorods. (**a**) SEM on silver with a magnification of ×50,000. (**b**) EDX on silver (**c**) SEM on silver/gold with a magnification of ×50,000. (**d**) EDX spectra on silver/gold.

Silver and gold nanoparticles with a very small size covering the silicon rods with an average length of 0.5 μm were observed on the surface by scanning electron microscopy (SEM), see Figure 2a,c. Energy-dispersive X-ray analyzer (EDX) scanning was performed on the surface of Ag and Au/ag deposited silicon nanorods, see Figure 2b,d. We were able to determine the percentage of each component of the surface. We found the most important components, such as 18% of silicon, 39.7% of silver, and 2.5% of gold. The covered nanostructures are 3D multi-oriented. The latter support them as active substrates for SERS experiments.

### 3.2. Testing Substrate’s Enhancement

**Figure 3:**
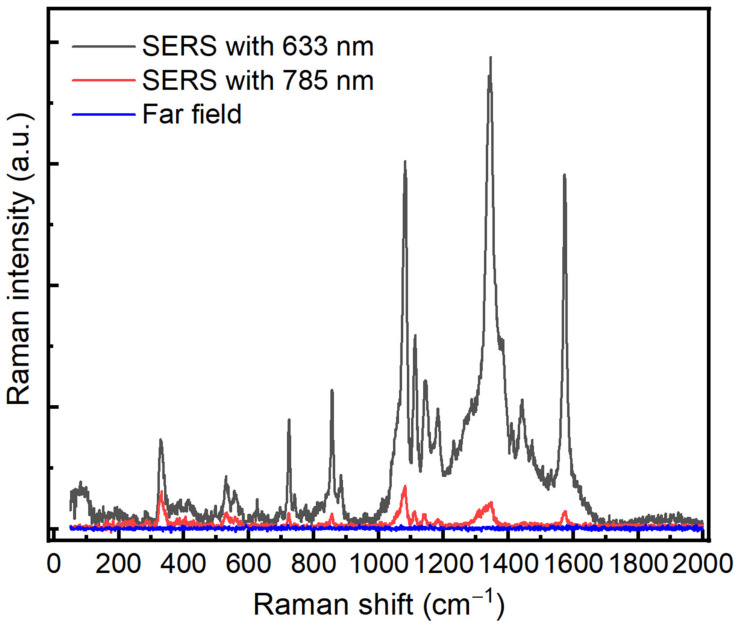
SERS spectra of 4-NTP on Au/Ag substrate with different excitation wavelengths, 632.8 nm (black color) and 785 nm (red color). Blue spectrum represents the Raman spectrum of 4-NTP on Au/Ag where there is no enhancement. Both wavelengths, 785 nm and 632.8 nm, are used. An integration time of 1 s, power 0.2 mW, ×50.

In order to test the optical enhancement of our prepared substrate, we have used 4-Nitrothiophenol (4-NTP) as a standard active probe molecule at two excitation wavelengths, 632.8 nm and 785 nm. In this regard, the glass control and Au/Ag substrates were immersed in a 10^−3^ M solution of 4-NTP prepared in ethanol absolute. The immersion was kept for 24 h to ensure a complete binding between 4-NTP and the Au/Ag substrate via the thiol group. The substrates were rinsed with ethanol to remove the non-bonded molecules and then used for SERS measurements. Figure 3 shows SERS spectra with the two different excitation wavelengths, 632.8 nm and 785 nm.

The SERS signals display the typical features of 4-NTP centered at 1080, 1350, and 1580 cm^−1^ corresponding to CH bending, NO_2_ symmetric stretching, and C–C stretching, respectively [20]. On the other hand, the different SERS results indicate that the spectrum at 632.8 nm exhibited a greater optical enhancement compared with the 785 nm laser wavelength. In order to avoid any effect generated from the far field, we have carried out a far-field measurement of 4-NTP on the substrate where we did not observe any enhancement by using the same conditions. The SERS enhancement factor of the Au/Ag substrate was estimated to be of the order of magnitude of 10^6^–10^7^ for 632.8 nm and 10^2^–10^3^ for 785 nm, respectively; see the Supplementary data. Our 3D simulation model also confirms these observed values. Based on these values, we concluded that 632.8 nm is the suitable wavelength for protein detection. 

### 3.3. Far Field and SERS Comparison on RBD

**Figure 4:**
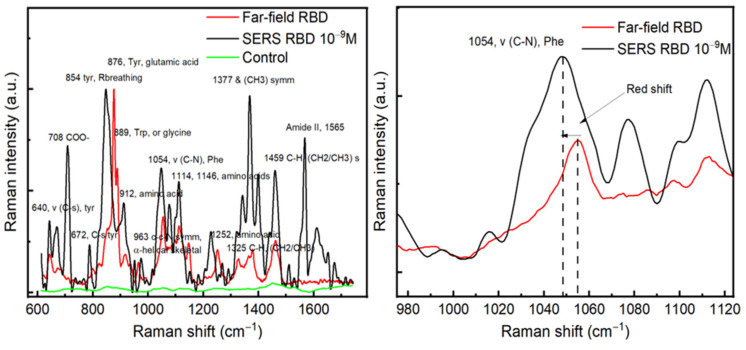
(**a**) A comparison between SERS and far-field of RBD, the green curve represents the Raman spectrum carried out on the Au/Ag substrate (control) without RBD proteins. (**b**) A zoom in on the peak position located around 1050 cm^−1^, a red shift is observed between SERS and Far-field spectra.

After achieving the optical enhancement with our substrate and finding the optimum excitation wavelength, SERS and far-field measurements were conducted on RBD protein. RBD was chosen in this study because of its conserved conformational-epitopes and its potential applications as a target for SARS-CoV-2 detection and RBD-based antibody assays for serology [6,7,8,9]. For the SERS analyses, a drop of 10^−9^ M from the RBD solution was deposited on the Au/Ag substrate and kept for complete dryness before measurements. However, for far-field spectra, the Raman measurements were conducted directly on a drop of 10 μL of protein (10^−5^ M) deposited on the CaF_2_ substrate. Figure 4 displays a comparison between SERS and far-field spectra obtained on RBD, where we can observe a red shift in the main SERS signals compared with the Raman far-field. In both cases, we detected the vibrational modes of the RBD protein. For example, a red shift of the peak located at 1054 cm^−1^ of the phenylalanine around 5 cm^−1^ is observed, see Figure 4a. The red shift in SERS relative to far-field is mainly due to the change of the localized plasmons depending on the dielectric functions of metallic nanostructures that, in turn, depend on different parameters, such as the medium, size of nanoparticles, and intrinsic damping of metal, which are known to display this interesting common phenomenon. Indeed, upon optical excitation, the maximum SERS enhancements appear at lower energies than the maximum of the corresponding far-field spectrum resulting directly from the physics of a driven and damped harmonic oscillator [21]. Our finite element simulation confirms this plasmonic origin.

The SERS spectrum allowed us to identify different peaks in the Raman spectra database of proteins [22,23]. The most prominent peaks in the SERS spectrum were located at 708, 854, 1054, 1114, 1377, 1459, and 1565 cm^−1^. The peak positioned at 708 cm^−1^ is mainly related to the O–O stretching in oxygenated proteins [24]. The peaks located at 1054 and 1114 and 1459 cm^−1^ can be attributed to the C–N signal in phenylalanine, C–C stretching, and the CH_2_ bending mode [25,26]. The peaks of great interest are located at 854 and 1377 cm^−1^, which correspond to the Tyrosyl residues, CH3 sym mode [27]. The amide II band (1520–1570 cm^−1^) appearing in SERS is primarily related to N–H bending, C–N stretching, and C–C stretching and is known to be very weak in non-resonance Raman spectra, which makes them nearly undetectable [28]. In terms of amino-acid assignment, the most prominent amino-acids observed in the SERS spectrum of RBD are serine or tyrosine (854 cm^−1^), threonine (876 cm^−1^) or glutamic acid (876 cm^−1^), phenylalanine (1054 cm^−1^), and lysine (1141 cm^−1^) [22,29]. 

### 3.4. Effect of RBD Concentration on the SERS Signal and Specific Study

**Figure 5:**
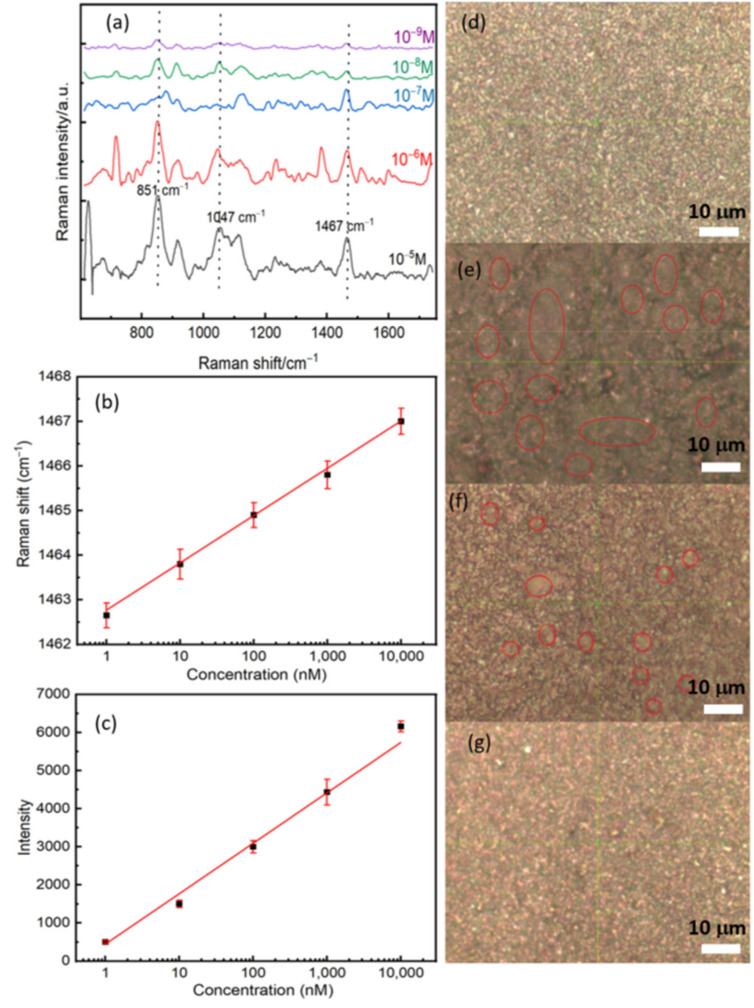
(**a**) The concentration-dependent SERS study and the concentration of RBD between 10^−5^ M and 10^−9^ M. (**b**) A semi-log plot of the Raman shift as a function of the concentration of RBD. (**c**) A semi-log plot of the intensity as a function of the concentration of RBD. Plotted data were extracted by fitted the Raman line located at 1467 cm^−1^ by using a Gaussian function. (**d**–**g**) Optical images with a 100× objective for three concentrations: (**d**) Control, (**e**) 10^−5^ M, (**f**) 10^−7^ M, and (**g**) 10^−9^ M. Red circles represent the position of the protein, and it is shown clearly the decreasing of protein number with the concentration.

After measuring the Raman far-field and optimizing the experimental parameters, we have performed a concentration-dependent SERS study by dropping different concentrations of RBD protein on the surface of the substrate. The optical images with the 100× objective showed an apparent decrease in protein agglomeration on the substrate with protein dilution (Figure 5d–g). The regions that contain the proteins seem like gray clouds. The SERS analyses of the dry spots showed a significant change in the feature of the Raman spectra by decreasing the concentration (see Figure 5a). As expected, the whole Raman intensity decreases, which can be correlated with the reducing molecules’ numbers by a surface unit. Since the protein is enormous, the concentration affects the reorientation of the molecular springs as with the plasmonic surface, giving rise to significant perturbations on the SERS spectra of the molecular springs, such as shifts and broadness of the vibrational features [30]. In order to quantify the shift, we fitted the vibrational mode located at 1467 cm^−1^ by using a Gaussian function. A semilog plot of the shift as a function of the concentration shows a good correlation with a constant slope between the shift and the concentration, see Figure 5b. We observe a red shift of the order of 4 cm^−1^ by decreasing the concentration from 10^−5^ M to 10^−9^ M. This red shift can be explained by the relaxation of tensile stress due to the interaction between proteins at higher concentrations. Figure 5c also shows a linear dependence between the Raman intensity and the concentration. The latter confirms the linear response property and sensitivity of our biosensor. 

**Figure 6:**
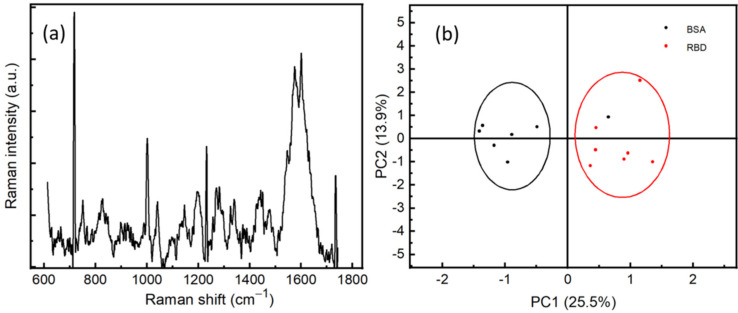
(**a**) SERS spectrum of RBD with a concentration of 10^−12^ M, averaged from 5 positions. (**b**) Principal component analysis of BSA (10^−9^ M) and RBD (10^−9^ M).

The most important observation here is that we could detect the Raman signal at a very low concentration of 10^−12^ M (Figure 6a). The lowest limit of detection we reached (10^−12^ M), which corresponds to 10 molecules of RBD protein in a laser spot of approximately 1 μm^2^. If each COVID-19 virus contains 100 RBD protein (trimers), our detection limit is of 10^−15^ M that corresponds to 1 virus per 10 μm^2^. The lowest detection limit we obtained with the SERS technique can emulate that of RT-PCR sensitivity which can approach 3.8 copies/mL depending on the used kit [31]. However, RT-PCR is affected by contamination and requires high-cost equipment and reagents. Further tests are required using actual COVID-19 positive samples to verify the ability of the presented SERS in detecting viruses [32,33,34].

In order to assure the specificity of our method of detection, we performed SERS measurements on RBD and BSA proteins at a concentration of 10^−9^ M. The measurements were carried out at different positions on the surface of the sample. We can observe the difference between the spectra of RBD and BSA, which can be explained by the fact that the vibrational modes of the proteins depend strongly on the number and conformational structure of amino acids existing within the proteins, see Figure S1. This difference correlates to the vibrational modes of the protein, which gives rise to a protein fingerprint. That indicates it is possible for SERS spectra to identify not only different virus species but also different strains within the same species. A statistical study has been performed on the Raman spectra of two proteins using principal component analysis (PCA), see Figure 6b. Preprocessing steps of treatment before the PCA, such as background subtracting, smoothing, and normalizing, have been performed. We can observe that the RBD proteins are located in the PC1 more than 0, while most of the BSA proteins are in the PC1 less than 0. This clearly shows the sensitivity and specificity of the SERS technique to discriminate between RBD protein and other proteins, such as BSA, at a very low concentration of 10^−9^ M.

### 3.5. Indirect Detection of RBD by Using SARS-CoV-2 RBD Antibody

**Figure 7:**
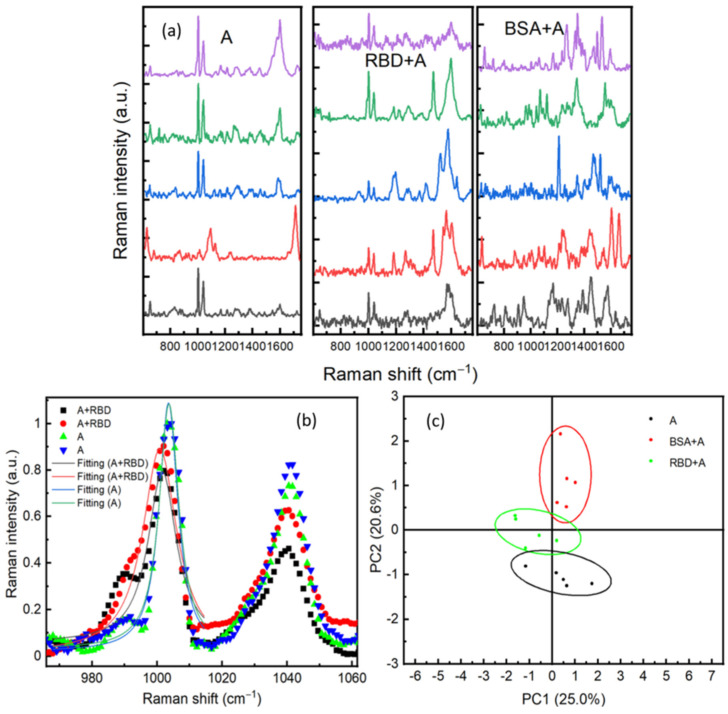
(**a**) SERS spectra performed on different positions respectively on antibodies, RBD on antibodies (RBD + A), and bovine serum albumin (BSA). (**b**) A comparison between SERS spectra of RBD + A and A in two positions; fitting of the Raman line with a Lorentzian function of RBD + A at 1001 cm^−1^ and A at 1003 cm^−1^. (**c**) Principal component analysis of SERS spectra for Antibody, RBD, and BSA.

**Figure 8:**
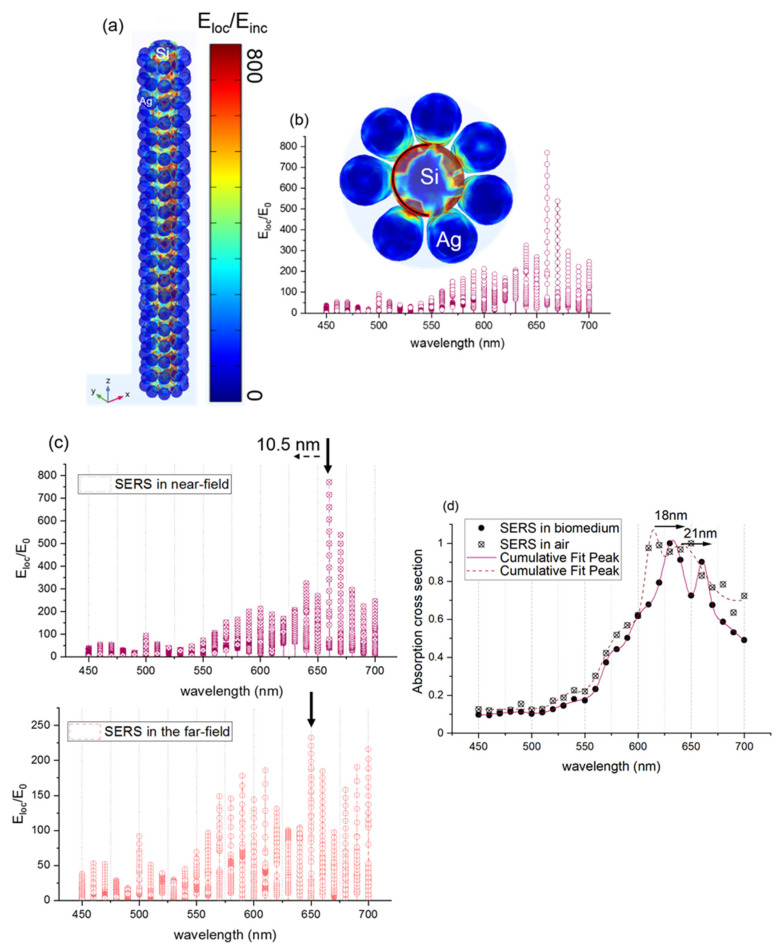
(**a**) 3D simulation of the electromagnetic field enhancement (near-field enhancement) in the silicon wire (10 nm radius) surrounded by a silver spherical nanoparticle (7 nm radius). The model is initially placed in the air domain. (**b**) Electromagnetic near-field enhancement (E_loc_/E_0_) collected in the silicon nanorod edge as a function of wavelength (**c**) Electromagnetic near-field enhancement (E_loc_/E_0_) of 3D silicon wire surrounded by nanospherical silver (7 nm radius) at a variable wavelength in the near-field and in the far-field. (**d**) Absorption cross-section of the 3D silicon nanowire surrounded by silver nanosphere-proteins.

In order to increase the selectivity of our biosensor, we have considered an antibody-antigen recognition on the surface of the Au/Ag substrate. This method should reduce the interferences of different signals generated from other proteins existing in the sample. The surface was activated with an RBD antibody (ARS-CoV-2 Spike Neutralizing Antibody). The SERS substrate was incubated for 24 h in sterile water containing 10^−8^ M of antibody. Following this, the substrate was rinsed with sterile water. The antibody was physically adsorbed on the SERS surface, and its presence was confirmed in SERS. To verify the specificity of the antibodies, 1 μL of either RBD or BSA (both prepared at 10^−9^ M) was dropped on the surface. After 20 min of drying, SERS measurements were performed by scanning different positions on the surface. Figure 7a shows SERS spectra performed on areas containing only antibody (A), RBD protein +antibody (RBD + A), and BSA protein +antibody (BSA + A). An important feature in (A) and (RBD + A)’s SERS spectra is the two intense bands observed at 1003 cm^−1^ and 1041 cm^−1^, which are assigned to the amino-acid phenylalanine [29]. Phenylalanine is an aromatic amino acid and considered a fundamental constituent of proteins. This SERS observation indicates that the aromatic group of phenylalanine is perpendicularly oriented toward the substrate. Indeed, in the SERS technique, a mode is highly enhanced if the polarizability component is perpendicular to the surface of the substrate [35]. Therefore, the increase in the band intensity could be related to the interaction of the C–N stretching band of this amino acid and the surface of the substrate [26]. Another important feature was the redshift of these vibrational modes located around 1003 cm^−1^ and 1041 cm^−1^ after adding RBD protein, see Figure 7b. The two peaks are shifted around 2 cm^−1^. This shift could be related to the interactions between the RBD protein and the antibodies. Redshift in the SERS spectra in response to an antibody-protein recognition is already reported exhibiting exceptional sensitivity and specificity [36]. Another explanation of this shift could be due to the change of the medium refractive index, as mentioned by our 3D simulation in Figure 8d. To verify the specificity of the presented detection method, principal component analysis (PCA) has been used for statistical analysis of Raman spectra of the three components (A, RBD + A, and BSA + A). As shown in Figure 7c, all RBD protein spectra are located in PC2 less than 0, whereas BSA is in PC2 more than 1. The convergence of spectra of antibody only (A) and RBD +A indicates the specificity of the antibody for RBD. The spacing between spectra of the antibody only, RBD + A, and BSA + A clearly shows the sensitivity and specificity of SERS to discriminate different proteins at a very low concentration of 10^−9^ M. Compared to the PCA of BSA and RBD only (Figure 6b), using the antibody is not essential for just purely SERS discrimination between different proteins.

## 4. Simulation

COMSOL Multiphysics software is used to execute expanded simulations and to investigate, in particular, the wave-optic interface covering the modeling of electromagnetic fields and waves in the frequency domain. We proceed with the interface by formulating firstly Maxwell’s equation (Equation (1)):

(1) $$∆\times \mu_{r}^{-1}\left(\nabla \times \vec{E}\right)-k_{0}^{2}\left(\varepsilon -\frac{j\sigma }{\omega \varepsilon_{0}}\right)\vec{E}=0$$

where ω, $\mu_{r}$, ε, and σ are the excitation frequency, the relative permeability (fixed to 1), the relative permittivity, and the electrical conductivity, respectively. $\varepsilon_{0}$ and $k_{0}$ represent, respectively, the permittivity and the wave number in free space, being $k_{0}=\omega /c_{0}$ (with $c_{0}$ the speed of light in a vacuum). In these simulations, we consider the relative dielectric permittivities that correspond to the optical frequencies (refractive index model with *n* and *k* real and imaginary refractive indexes, respectively, for the electric displacement), ε = (*n* − i*k*)^2^ (in Equation (1)). Finally, we suppose a perfect matched layer that absorbs the propagation of the wave within the computational region and considering reflections in the interior interface. We use the finite element method to solve and discretize the equation in numerically stable edge elements.

The simulation is performed with the same experimental conditions for the sake of precise prediction of the electromagnetic field distribution in the three-dimensional (3D) model of silicon nanorod surrounded with Ag spherical nanoparticles. We were limited to the use of silver nanoparticles since they are dominant with respect to gold.

The mechanism of electromagnetic field enhancement can be resumed in two steps as follows: The electromagnetic enhancement can be generated first in the vicinity of the Ag nanoparticles where the polaritons are formed. The local field is further amplified, and a dipole is produced conducting to the enhancement of the Raman scattering in the nanogap and a formation of plasmons [37,38,39,40]. After, an interactive excitation from the system of the Ag nanoparticles at a resonant frequency (plasmon resonance) generates an enhanced apparent Raman polarizability. Consequently, the calculated enhanced Raman scattered light *G* from the structure of silicon nanorod surrounded by Ag nanoparticles (Equation (2)) is presented as follows:

(2) $$G=\left(\frac{I_{SERS}}{I_{Raman}}\right)\times \left(\frac{N_{Raman}}{N_{SERS}}\right)$$

where $I_{SERS}$ is the intensity of SERS generated by Ag nanoparticle, $I_{Raman}$ is the intensity of Raman far field generated from silicon nanorod, $N_{Raman}$ is the number of molecules in a laser spot generated the far-field, and $N_{SERS}$ is the number molecules generated SERS signal. $N_{SERS}$ is estimated by taking into account the surface area of the 3D silicon nanorod surrounded by Ag nanoparticles.

Figure 8a presents the electromagnetic field enhancement (E_loc_/E_inc_) in the silicon nanorod surrounded by the spherical silver nanoparticles (7 nm radius of silver and ≤5 nm interparticle distance in a longitudinal direction). The configurations in the xyz plane and in the xy plane are presented to show better the localized field enhancement. The color bar on the right presents the strength of Eloc/Einc. Therefore, the red color means that Eloc/Einc is very high, and the blue color indicates that it is very low. Figure 8b shows that the maximum field enhancement is Eloc/Einc = 800, this leads to SERS enhancement factor (Eloc/Einc)^4^ = 4.10^11^, and it is located both between the spherical silver nanoparticles and between the silicon nanorod and the silver spherical nanoparticles. The latter confirms the high optical enhancement of metallic nanostructures observed by testing the probe molecules of 4-NTP by using 632.8 nm. Figure 8c depicts the enhancement factor of the SERS substrate formed by the silicon nanorod and the silver spherical nanoparticles in the far-field and in the near-field. A clear redshift of about 10.5 nm of the plasmon resonance around 695 nm in the near field is detected. This result is in great agreement with the experimental redshift observed in Figure 4. In order to study the effect of the proteins (RBD) on the SERS substrate, we simulated the SERS substrate with a mixture of proteins that increases the refractive index of the initial medium. Figure 8d presents the simulated SERS substrate in air and SERS substrate in a medium that contains proteins (biomedium). Two plasmon resonance peaks are detected, and they are located at 615 nm and 640 nm for SERS substrate in air. Those two plasmon resonance peaks are shifted by 18 nm and 21 nm, respectively, as proteins are placed on the SERS substrate and lead to the same experimental shift observed in Figure 7b. Therefore, the SERS frequency shift sensing has a potential application for trace proteins detection.

## 5. Conclusions

In this work, we developed a new approach based on surface-enhanced Raman spectroscopy (SERS) as a simple, fast, and accurate technique for early SARS-CoV-2-RBD detection. We have successfully fabricated Au/Ag nanostructures covered by silicon nanorods that showed higher optical enhancement. The latter showed a linear response property by varying the concentration of RBD protein. Its sensitivity was confirmed by the detection of RBD protein at a very low concentration of 1 pM and during a short time of 3 sec. Besides, the SERS substrate was able to discriminate the RBD protein from other proteins such as BSA, confirming this technique’s fingerprint specificity. By changing the way of detection, we successfully detected RBD protein from other proteins such as BSA indirectly by using an antibody. A statistical study has been performed by taking different SERS spectra confirmed the specificity and selectivity of our approach. A red shift in the Raman bands due to the interaction between the RBD protein and its antibody was also observed. Finally, we find a very good agreement of the plasmon shift and near-field enhancement obtained in experiments and in the simulated 3D model. 

Further studies on actual specimens are required to verify the applicability of using SERS for virus detection. The proposed methodology will pave the way to detect the COVID-19 virus in a short time and with no prerequest treatment. There is a high potential to apply Raman for detecting pathogens in real-time. 

## Data Availability

Not applicable.

## Supplementary Materials

The following are available online at https://www.mdpi.com/article/10.3390/s21134617/s1, Figure S1: SERS spectra of RBD and BSA. Figure S2: Simulation of enhancement factor. Figure S3: SEM image of Au/Ag covered silicon nanorods with lower magnification.
