# Supplementary material for: SARS-CoV-2 Receptor Binding Domain as a Stable-Potential Target for SARS-CoV-2 Detection by Surface—Enhanced Raman Spectroscopy
Source: Sensors (Basel). 2021 Jul 5;21(13):4617. doi: 10.3390/s21134617 (PMC8271591; doi:10.3390/s21134617)
Supplement: Supplementary file 1 [file sensors-21-04617-s001.zip › sensors-1237772-supplementary.pdf]

SARS-CoV-2 receptor binding domain as a stable-potential target for SARS-CoV-2 detection by surface –enhanced Raman spectroscopy

Chawki Awada[1,*], Mohammed Mahfoudh BA Abdullah[2], Hassan Traboulsi[3], Chahinez Dab[4] and Adil Alshoaibi[1]

[1] Department of Physics, College of Science, King Faisal University, P.O. Box: 400, Al-Ahsa 31982 and Saudi Arabia.
[2] Department of biological sciences, College of Science, King Faisal University, P.O. Box: 400, Al-Ahsa 31982, Saudi Arabia.
[3] Department of Chemistry, College of Science, King Faisal University, P.O. Box: 400, Al-Ahsa 31982, Saudi Arabia.
[4] Département de chimie, Université de Montréal, Montréal QC H3C 3J7, Canada.
* Correspondence: author: cawada@kfu.edu.sa, tel: +96635899533

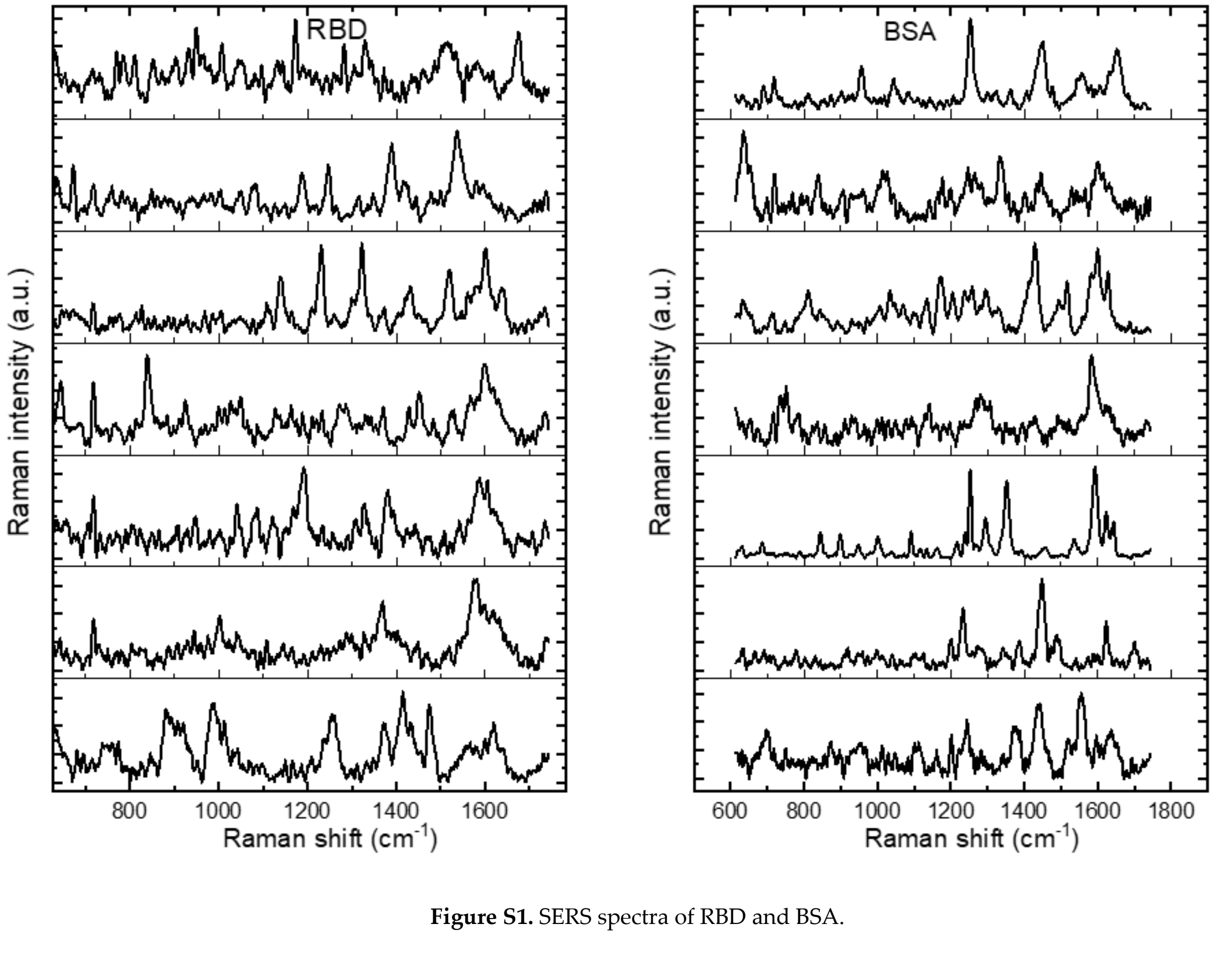

Figure S1. SERS spectra of RBD and BSA.

Estimation of Enhancement factor:

$$G = \frac{I_{SERS}}{I_{Raman}} \frac{N_{Raman}}{N_{SERS}}$$

with I_{SERS} is the intensity of SERS generated by 4-NTP on Au/Ag nanoparticles, I_{Raman} the intensity of Raman far field generated from SERS substrate where there is no enhancement. N_{Raman} is the number of molecules excited by a laser spot with a diameter of 1.5 µm. N_{SERS} is the number of molecules excited by the hotspots between nanoparticles. As the

occupied surface area of a molecule is 0.5 nm2 and the surface of laser spot is around 1.8 µm². Therefore, N_{Raman} is approximately 106 molecules. It is important to mention that the number of hotspots excited by the gaps N_{SERS} is much less than N_{Raman}. The latter depends on the surface density of nanoparticles and also the density of hotspots. Based on different statistical study reported in our previous work (Awada et al., A. High resolution scanning near-field mapping of enhancement on SERS substrates: comparison with photoemission electron microscopy. Phys. Chem. Chem. Phys. 2016, 18, 9405-9411). If we roughly estimate a number of 10^2-10^3 excited molecules, thus $\frac{N_{Raman}}{N_{SERS}} = 10^3 - 10^4$. From Figure 3, $\frac{I_{SERS}}{I_{Raman}} = 400$ for λ=632.8 nm and 20 for λ=785 nm, hence, the enhancement factor square is respectively 10^6-10^7 and 10^2-10^3 for 632.8 nm and 785 nm. The values match well with those obtained by simulation, see figure 8.

According to the enhancement factor presented in the simulation, we can confirm that the use of only silicon wire (10 nm radius) leads to a maximum enhancement factor equal to 90. However, the use of only silver nanoparticles (7 nm radius) without a silicon wire leads to a maximum enhancement factor equal to 325. In these two figures, we used the same model's dimension used in figure 8 (a) to better highlight the difference of using only silicon wire, only silver nanoparticles and combined silver and silicon wire presented in figure 8 (a) with a maximum enhancement factor equal to 800.

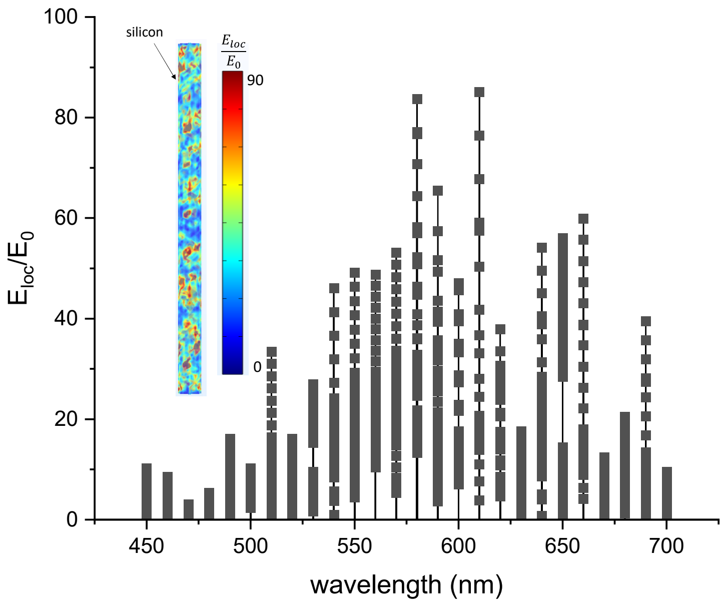

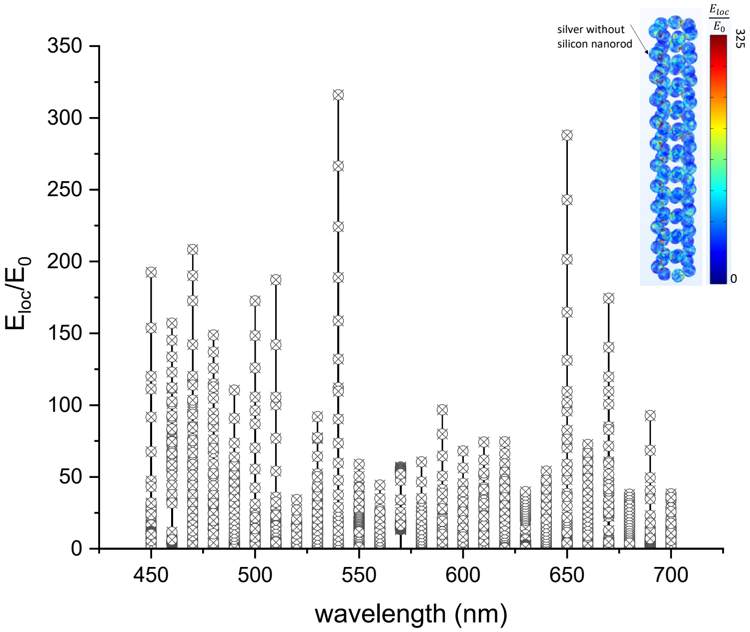

Figure S2. Simulation of enhancement factor.

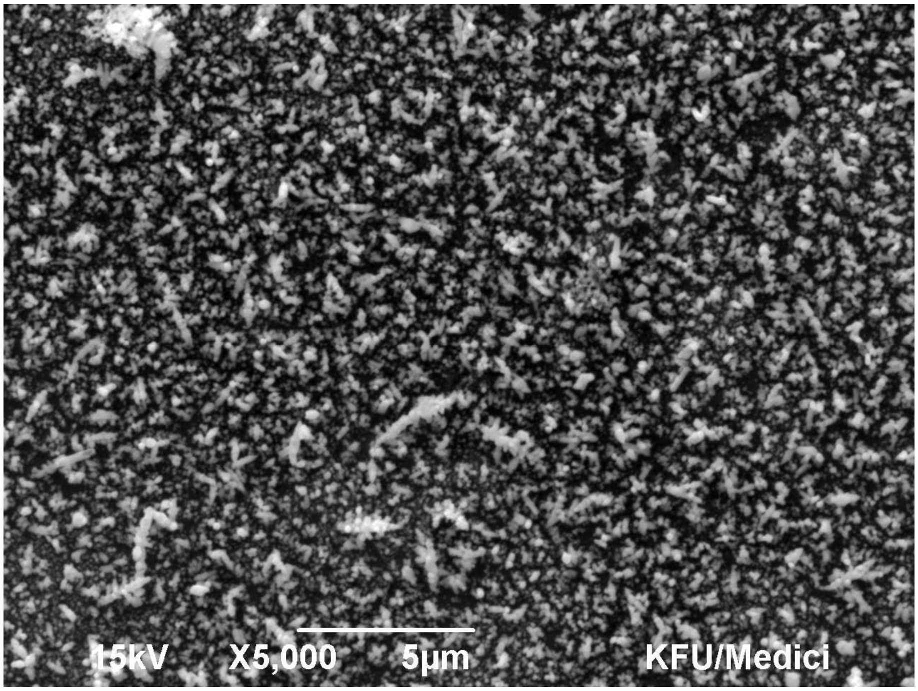

Figure S3. a SEM image of Au/Ag covered silicon nanorods with lower magnification.
